# Extracellular Vesicles in Bone Homeostasis: Emerging Mediators of Osteoimmune Interactions and Promising Therapeutic Targets

**DOI:** 10.7150/ijbs.69816

**Published:** 2022-06-21

**Authors:** Xiaoyuan Huang, Yanhua Lan, Jiahui Shen, Zhuo Chen, Zhijian Xie

**Affiliations:** Stomatology Hospital, School of Stomatology, Zhejiang University School of Medicine, Clinical Research Center for Oral Diseases of Zhejiang Province, Key Laboratory of Oral Biomedical Research of Zhejiang Province, Cancer Center of Zhejiang University, Hangzhou 310006, China.

**Keywords:** Extracellular vesicle, Exosome, Osteoimmune interaction, Bone disease

## Abstract

An imbalance in bone homeostasis results in bone loss and poor healing in bone diseases and trauma. Osteoimmune interactions, as a key contributor to bone homeostasis, depend on the crosstalk between mesenchymal stem cell-osteoblast (MSC-OB) and monocyte-macrophage (MC-Mφ) lineages. Currently, extracellular vesicles (EVs) are considered to be involved in cell-to-cell communication and represent a novel avenue to enhance our understanding of bone homeostasis and to develop novel diagnostic and therapeutic options. In this comprehensive review, we aim to present recent advances in the study of the effect of MC-Mφ-derived EVs on osteogenesis and the regulatory effects of MSC-OB-derived EVs on the differentiation, recruitment and efferocytosis of Mφ. Furthermore, we discuss the role of EVs as crucial mediators of the communication between these cell lineages involved in the development of common bone diseases, with a focus on osteoporosis, osteoarthritis, bone fracture, and periodontal disease. Together, this review focuses on the apparent discrepancies in current research findings and future directions for translating fundamental insights into clinically relevant EV-based therapies for improving bone health.

## Background

Osteoimmunology is an emerging field exploring immune processes and bone metabolism 1. Bone cells (bone mesenchymal stem cells (BMSCs), osteoblasts (OBs), osteoclasts (OCs), and osteocytes), immune cells (T cells, B cells, neutrophils, and macrophages (Mφ)), hematopoietic stem cells (HSCs), and myeloid and lymphoid progenitor cells share the same micro-environment, this leads to them sharing a variety of molecules, functioning together as osteoimmunology. BMSCs, known as “universal cells”, have self-renewal capacity and multiple differentiation potency into OBs, chondrocytes, and adipocytes 2. In pathological circumstances, significant unbalanced differentiation of BMSCs into adipocytes/chondrocytes occurs. Mφ as dynamic cells, derived from monocytes (MCs), participating in induction and resolution of metabolic/inflammation exhibit a significant degree of plasticity and heterogeneity through their function and biology. As important components of osteoimmunology, BMSCs and Mφ with same characteristic-remarkable genotypes, cellular phenotypes, and function plasticity sit at the center of a complex and tightly regulated system. Moreover, the intimate interactions between BMSC-OB and MC-Mφ are fundamental in maintaining bone homeostasis, as illustrated by the activation of tissue repair Mφ (M2) upon promoting BMSC osteogenic differentiation, a process that is impaired in most bone loss diseases (e.g. osteoporosis and periodontitis).

**Figure 1:**
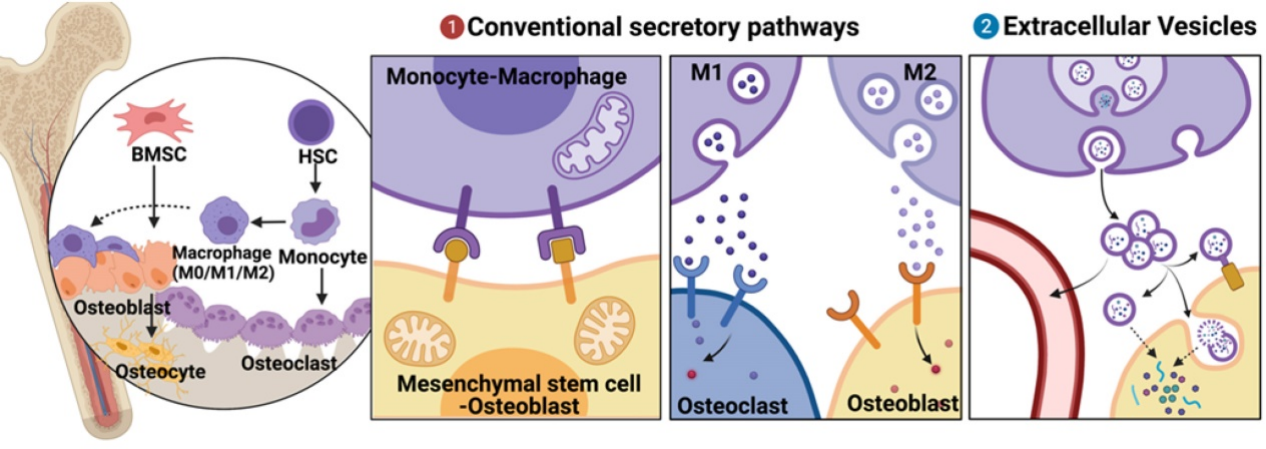
** Brief description of mesenchymal stem cell-osteoblast (MSC-OB) lineage and monocyte-macrophage (MC-Mφ) lineage in bone tissues and their different modes of communication. (a)** Conventional secretory pathways (including direct cell-cell contact and chemical receptor-mediated events) and **(b)** extracellular vesicles (EVs). Schematic picture was created with BioRender.

Mediators of osteoimmune interactions can be divided into two groups, namely, (i) conventional secretory pathways, including direct cell-cell contact and chemical receptor-mediated events (such as those involving cytokines and other chemical mediators) and (ii) extracellular vesicle (EV)-mediated events. The mechanisms of the former interactions have been thoroughly studied and elucidated 3, 4 **(Fig. 1)**. However, EVs, which were considered only to be “garbage disposal” systems; are currently being increasingly recognized as playing important roles in short- and long-distance intercellular communications 5.

EVs are small vesicles of 30-150 nm in diameter with a lipid bilayer. Following the release of EVs by exocytosis from cells, they interact with the target cells and transport intracellular components including proteins, lipids, messenger RNAs (mRNAs), and microRNAs (miRNAs) to the cytosol of target cells via endocytosis 6. EVs can also mediate signal cascades with secretory cells and the extracellular matrix (ECM) or can be released into the blood and lymphatic vessels to function in long-distance communication 7, 8. As a cell-free therapeutic strategy for bone-related diseases and bone repair, EVs possess unique advantages such as nano size, non-toxicity, low immunogenicity, biocompatibility, and flexibility of use, thereby garnering attention 9. There is now broad consensus that EVs are part of the intercellular signaling network that takes place in osteoimmunology. However, most studies were focused on two primary areas: intrinsic regulation in a single cell lineage (osteocyte, BMSCs, and osteoprogenitor) or cell-to-cell traffic in coupling of angiogenesis and osteogenesis. A comprehensive review of EVs-mediated regulation between MC-Mφ and MSC-OB is lacking.

Moreover, in terms of the mechanisms in communication between MC-Mφ and BMSC-OB via EVs, the most contentious aspect, at this time, are the functions of EVs derived from different types of Mφ on recipient MSC-OB cells. It should also be highlighted that regarding EVs derived from MSC-OB cells, impacting Mφ polarization should not always be seen as their main function; the other functions of these EVs (e.g., recruitment, efferocytosis, differentiation) are worth being known, despite being relatively understudied. The purpose of this article is to elucidate the crosstalk between MC-Mφ and MSC-OB in multiple ways, highlight cell-to-cell communication by EVs, cover advances in research on EVs derived from the MSC-OB lineage and MC-Mφ in bone disease. Finally, we discuss areas that are the most contentious and propose areas that are in particular need for further research in bone homeostasis.

## MSC-OB regulation by MC-Mφ via EVs

MC-Mφ affect the proliferation, differentiation, recruitment, survival, function of MSC-OBs, and ultimately, bone homeostasis. MC migrates from the circulation to the local tissue and differentiates into Mφ, which plays specific roles. Mφ as highly heterogeneous and plastic cells can, depending on the stimulus and microenvironment, polarize into different types *in vivo*, including M0, M1, M2. Non-activated M0 can be classically activated by prostaglandin E2 (PGE2), tumor necrosis factor (TNF)-α, interleukin (IL)-1β, and IL-6, which initiate the immune response and remove pathogens and tumor cells 10, 11.

Alternatively, the anti-inflammatory M2 phenotype is activated by IL-4, IL-13, or IL-10 and expresses high levels of arginase 1 (Arg1), IL-10, IL-1ra, and cluster of differentiation 206 (CD206), which plays a central role in tissue repair and neovascularization 4, 12. In addition to Mφ, OC, which are also derived from the hematopoietic mononuclear precursor cells, can be stimulated by Mφ colony-stimulating factor (M-CSF) and receptor activator of NFκB ligand (RANKL) produced by OB to differentiate into bone resorptive cells 13. These tissue-resident Mφ line the bone surface and contribute to osteoclastic bone resorption 14. In this review, OC is included in the class of MC-Mφ according to their cell derivation.

### Regulation of MSC-OB by MC-Mφ through conventional secretory pathways

Direct cell-to-cell contacts and chemical receptor-mediated events are conventional, effective regulatory mechanisms between the MSC-OB and MC-Mφ. It is reported that the osteal Mφ efferocytose apoptotic OB via αvβ3 or Mer linking proteins milk fat globule factor (MFG)-E8 or growth arrest specific 6 (Gas6) 15. In addition, the direct contact between OB and OC involves interactions among ephrin B2(EFNB2)-ephrin type-B receptor 4 (EPHB4), FAS-Fas ligand (FASL), and neuropilin 1(NRP1)-semaphorin(SEMA3A), and it regulates cell proliferation, differentiation and survival 16.

In chemical receptor-mediated event regulation, M1 respond to the inflammatory environment, promoting inflammatory osteoclastogenesis via M-CSF, TNF-α, IL-1β, IL-6, IL-17, and IL-23 secretion 17. M2 mediate the recruitment and differentiation of BMSCs by secreting CC motif chemokine ligand 2 (CCL2), CXC motif chemokine ligand 8 (CXCL8), stromal cell-derived factor 1 (SDF-1), and other chemokines.

Biomaterials can modulate Mφ polarization by inhibiting NFκB phosphorylation or by promoting the release of vascular endothelial growth factor (VEGF) and Arg1 19,20. These effects are mediated through the activation of bone morphogenic protein (BMP)-2/Smad and phosphoinositide 3-kinase (PI3K)/AKT signaling pathways, which facilitates bone tissue regeneration 18, 19. Thus, converting the Mφ phenotype from pro-inflammatory to anti-inflammatory by modulating cytokines and signaling pathways, eventually alter its function and its capability to regulate the direction of BMSC differentiation and promote osteogenesis.

### Effect of MC-Mφ-derived EVs on osteogenesis

**Figure 2:**
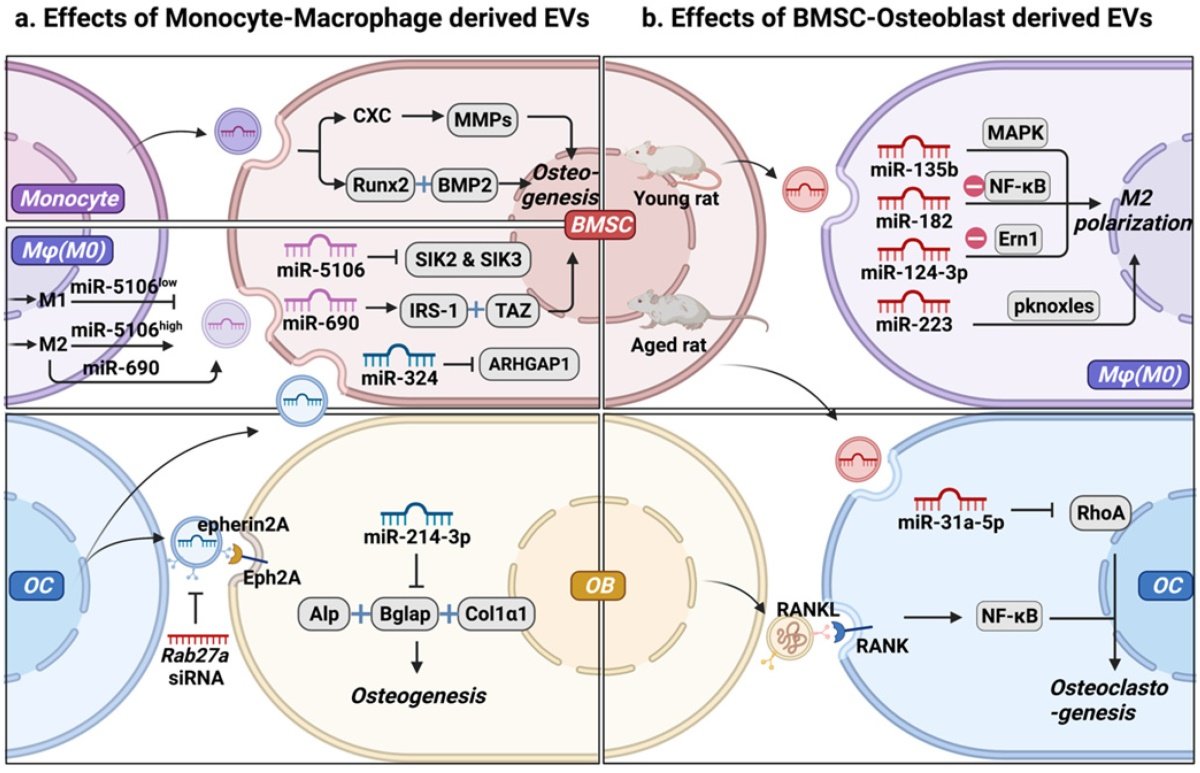
** Intercellular communication between mesenchymal stem cell-osteoblast (MSC-OB) lineage and monocyte-macrophage (MC-Mφ) lineage is mediated by extracellular vesicles (EVs). (a)** Effect of MC-Mφ-derived EVs on osteogenesis. **(b)** MSC-OB-derived EVs regulate monocyte-macrophages recruitment, polarization, and function. Schematic picture was created with BioRender.

EVs, as an important medium of intercellular communication, carry molecular cargo and transfer bioactive components **(Table 1** and **Fig. 2a)**. Signals activated in MC promote the differentiation of BMSCs into bone lining cells. Karin et al. 20 demonstrated that human MC-EVs improve the secretion of RUNX2 (RUNX family transcription factor 2) and BMP-2 (bone morphogenic protein-2) in hBMSCs, promoting osteogenic differentiation. Arjen et al. 21 reported that MC-EVs upregulated the expression of genes coding matrix metalloproteinases (MMPs) and increased the secretion of CXC, thereby stimulating processes related to the reorganization of the ECM structure, directing differentiation towards bone lining cells, which could differentiate into bone-forming OB. Interestingly, exposure in the inflammatory context may not undermines MC-EVs function in osteogenesis. Ekström et al. demonstrated that LPS-stimulated human MCs releasing EVs promoted the gene expression of the osteogenic markers (e.g., RUNX2, BMP2) 22. These findings indicate that the mechanisms by which MC-derived EVs promote osteogenesis are uncertain and require further investigation.

Mφ, as an essential component of the innate immune system, and bone marrow derived Mφ (BMM) is also closely associated with OB in the endosteal and periosteal surfaces. Furthermore, they present different phenotypes and functions in different microenvironments, and EVs derived from different Mφ phenotypes (M0-EVs, M1-EVs, and M2-EVs) play different roles in osteogenesis. Yu et al. 23 demonstrated that EVs secreted by different Mφ phenotypes could be internalized by BMSCs. Specifically, M1-EVs promoted the proliferation of BMSCs, with the highest expression levels of alkaline phosphatase (ALP), RUNX family transcription factor 2 (Runx2), osteocalcin (OCN), and BMP-2. In contrast, M2-EVs impaired the proliferation of BMSCs, whereas M0-EVs did not significantly influence the proliferation of BMSCs 23. However, the unique role of different polarized Mφ in osteogenesis has not yet been elucidated. Yuan et al. 24 demonstrated that miR-5106 is highly enriched in M2-EVs and that it can be transferred to BMSCs where it targets *SIK2* and *SIK3*, thereby accelerating bone remodeling. MiR-5106 expression reportedly decreased in M1-EVs, and M1-EV-treated cells exhibited similarly reduced mineral deposition and low levels of ALP compared with untreated cells 24. These findings indicate that EVs from the same phenotype Mφ have different effects on the osteogenic differentiation of BMSCs. M2-EVs increased the expression of miR-690, IRS-1, and TAZ in BMSCs, inhibiting adipogenesis and promoting osteogenesis of BMSCs 25. EVs with complex cargos have been suggested to play different roles in BMSC differentiation. Alternatively, different Mφ phenotypes regulate the osteogenesis of BMSCs through different signal pathways, depending on EVs and cytokines, separately or jointly 26.

OC functions as the only giant multinucleated cell, deriving from Mφ precursor and is mainly involved in bone resorption 27. OC participates in normal bone accrual, growth, and modeling, thereby maintaining bone metabolism through calcium metabolism and its lifetime integrity 28. OC-derived EVs (OC-EVs) contain multiple bone regulatory proteins that modulate OB formation 29. Huynh et al. 29 found that RANK-rich OC-EVs inhibit the formation of OC-like multinucleated cells by suppressing the interaction of RANKL-RANK 30. Interestingly, they found that OC-EVs, but not pre-OC-EVs, are rich in RANK, which promotes the differentiation of OC rather than the inhibition of OC-EVs in the formation of OC 30. Sun et al. 31 found that OC-EVs bound to OB through an EFNA2/EphA2 interaction to impair OB function by releasing miR-214. Li et al. 32 reported that OC-derived exosomal miR-214-3p could be transferred into OB to suppress bone formation. Liang et al. 33 found that OC-EVs were rich in miR-324, whereas ARHGAP1, which inhibits osteogenic differentiation, was downregulated and the miR-324-enriched EV-modified scaffold promoted bone regeneration. These observations suggest that OC can activate OB on the other side of the bone through OC-EVs, similar to the coupling factors that mediate cell-cell coupling. The mechanism by which OC-EVs influence bone remodeling still needs to be investigated to improve our understanding of the interaction between OB and OC as a crucial mechanism of maintaining cell homeostasis 34. In the bone microenvironment, EVs act as carriers of bioactive molecules that regulate osteogenesis through MC, modulate the Mφ polarization phenotype, and regulate OC, representing a new mode of intercellular communication. Subsequently, OC secretes EVs that carry different cargos, which differentially regulate osteogenesis.

## MC-Mφ regulated by MSC-OB via EVs

A dense spongy layer containing the commonly reported MSC-OB, including bone BMSC, OB, and osteocyte 35, serves as a barrier to protect the bone marrow. BMSCs have the potential for self-renewal and multidirectional differentiation 36. OB is derived from mesenchymal precursor, which is transformed to committed pre-OB following the expression of RUNX2 and Osterix. These cell lineages in the bone surface continue to differentiate into matrix-producing OB 37. Osteocytes are derived from OB embedded in the bone matrix and constitute over 90% of cells in the bone 38.

MC-Mφ are important immune cells in the bone marrow that are involved in inflammation and immune regulation, this suggests that in bone injuries or osteoarthritis, MSC-OB and MC-Mφ are tightly interrelated.

### Regulation of MC-Mφ by MSC-OB through conventional secretory pathways

Studies on MSC-OB to MC-Mφ have focused on their secreted soluble factors such as M-CSF and transforming growth factor (TGF)-β 39. A few studies have reported the direct cell-to-cell contact of MC-Mφ as target cells, and we found that most related studies have been based on BMSCs, and not on other types of MSC-OB 34. BMSCs have been shown to reciprocally modulate Mφ polarization phenotypes and regeneration via multiple signaling pathways, such as the NFκB, signal transducer and activator of transcription 3 (STAT3), and Akt pathways 39-41. BMSCs regulate Mφ recruitment and phenotypic polarization to promote tissue regeneration by secreting chemokines, cytokines, and other signaling factors 35. In conclusion, BMSCs attenuate injury and promote healing by modifying the polarization status of Mφ and suppressing their inflammatory reaction.

### MSC-OB-derived EVs regulate Mφ

BMSCs are multipotent stem cells found in the bone marrow and are attractive sources of regenerative medicine 42, 43. They have strong paracrine, anti-inflammatory, immunoregulatory, and angiogenic capabilities 44. In addition to the growth factors and cytokines produced by BMSCs, EVs have gained interest as intriguing signals that can shuttle payloads between cells. Recent studies have shown that the therapeutic benefits of BMSCs can be attributed to their EVs, and their use represents a potential cell-free strategy for osteogenesis 45. Moreover, BMSC-EVs could be powerful tools for Mφ recruitment, polarization, and functionalization 46 **(Table 2** and** Fig. 2b)**. BMSC-EVs promote the regeneration of periodontal tissues, partially through their involvement in regulating Mφ polarization and TGF-β expression to modulate the inflammatory immune response 47. Xu et al. 47 found that BMSC-EVs transformed Mφ subsets from the M1 to M2 phenotype* in vitro* under lipopolysaccharide (LPS) stimulation and reduced post-infarction inflammation by increasing M2 and degrading M1 polarization in the ischemic heart 48. In tendon-to-bone healing, Shi et al. 48 demonstrated that BMSC-EVs promoted fibrocartilage formation by increasing M2 polarization 49. In addition to identifying functional changes, studies have traced molecular mechanisms. For examples, Wang et al. showed that BMSC-EVs attenuated cartilage damage by carrying highly expressed miR-135b. This effect promoted M2 polarization of SMs by targeting mitogen-activated protein kinase (MAPK) 50. Thus, BMSC-EVs are crucial candidates for the immunomodulation of Mφ polarization. Zhao et al. 51 proved that BMSC-EVs attenuated myocardial ischemia-reperfusion by shuttling miR-182, which modified Mφ polarization through the Toll-like receptor 4 (TLR4)/NFκB pathway. Li et al. 3 demonstrated that exosomal miR-124-3p derived from BMSCs attenuated nerve injury induced by spinal cord ischemia/reperfusion injury (SCIRI) by regulating endoplasmic reticulum to nucleus signaling 1 (Ern1) and M2 polarization. However, the expression of miR‐31a‐5p was notably higher in BMSC-EVs from aged rats than in those from young rats. This effect increased the osteoclastic number and function by blocking E2F2 activity, resulting in SAHF assembly, and the dysfunction was reversed by miR‐31a‐5p antagomir 52, 53. Overall, we propose that MSC-EVs effectively modify Mφ polarization and promote cementogenic differentiation in the bone microenvironment. In turn, BMSC-EVs regulated M1 to M2 switching in Mφ and maintained the presence of M2 to promote tissue repair, thus forming a strong positive feedback effect.

Furthermore, MCs could differentiate into bone resorbing OCs, which are independent of Mφ polarization, as OCs differentiate preferentially with pro-inflammatory MCs 54, whereas inducers that replace activated Mφ inhibit OC osteocyte differentiation 55. Li et al. 56 demonstrated that EVs shed from OB containing the RANKL protein could be internalized by OC precursors via the stimulation of RANKL-RANK signaling to facilitate OC formation. A similar result was reported by Cappariello et al. 57, they found that OC-EVs interacted with OC precursors through the RANKL-RANK mechanism because RANKL^-/-^ EVs did not preserve OC functionality.

Despite the scarcity of studies, the few that exist appear to demonstrate the efficacy of MSC-OB-derived EVs in Mφ recruitment. Huang et al. found that in contrast to young MSC-EVs, aging MSC-EVs failed to alter Mφ phenotypes and reduce Mφ recruitment 58. After isolation of EVs of fibrodysplasia ossificans progressiva patients, analysis of the liquid chromatography/mass spectrometry results revealed that the heterotopic ossification occurs via the Ephrin B signaling pathway by aiding in Mφ chemotaxis and activation 59.

Apart from EVs secreted by viable BMSCs, apoptotic vesicles (apoVs) produced by apoptotic BMSCs have also been implicated in the regulation of Mφ functions. Zheng et al. 60 found that BMSC-derived apoVs could induce Mφ reprogramming in an exocytosis-dependent manner in the treatment of type 2 diabetes livers. Mechanistically, they demonstrated that proteins in BMSC-derived apoVs contribute to Mφ polarization towards the anti-inflammation phenotype. This phenomenon, however, has not yet been shown to occur in bone homeostasis. Given the increased fracture risk in adults with type 2 diabetes, future relative studies into the BMSC-derived apoVs applied in bone defect repair in diabetes mellitus will be of particular interest.

## EVs are crucial mediators between MSC-OB and MC-Mφ in bone disease

### Osteoporosis (OP)

**Figure 3:**
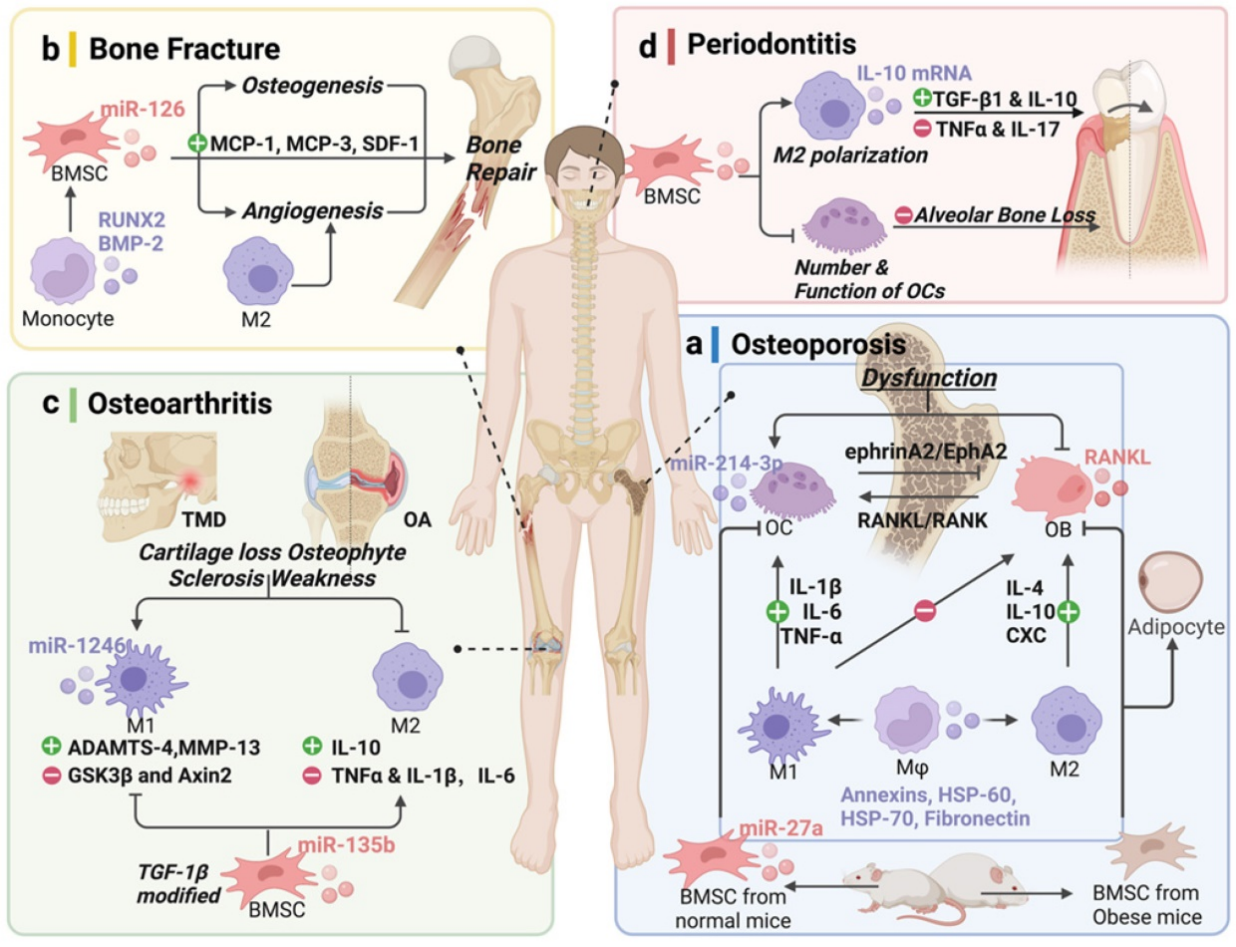
** Potential of extracellular vesicles (EVs) derived from bone-related cells and monocyte-macrophages in bone disease therapy. (a)** Osteoporosis (OP), **(b)** bone fracture, **(c)** osteoarthritis (OA), and **(d)** periodontal disease. Schematic picture was created with BioRender.

Osteoporosis (OP) is the most common metabolic bone disorder characterized by low bone volume and microarchitectural destruction, which leads to susceptibility to bone fragility and fracture 61, especially in the aging population 62. The dysfunctional activities of OB and OC are the primary causes of OP **(Table 3** and** Fig. 3a)** 63. OC, the only bone resorption cells, are also the current research priority. Li et al. 54 observed that miR-214-3p is rich in OC, and that its level in EVs increased. Furthermore, bone formation decreased in OC-specific miR-214-3p knock-in mice, and the OC-targeted RNA antagonist antagomir-214-3p reversed the inhibition in ovariectomized (OVX) mice 57. Sun et al. 64 found that OC-EVs specifically recognized OB through the interaction between EFNA2 (carried by OC-EVs) and EphA2 (on OB). In addition, miR-27a has been found to be more highly expressed in normal mice than in OVX mice, and that it interacts with Dickkopf WNT signaling pathway inhibitor 2 (DKK2) to reduce the number of OCs, while reversing OP symptoms 65. These results indicate that OC-EVs contain molecular cargo and regulate cell communication between OC and OB to inhibit bone formation.

Despite the imbalance between OB and OC, OP is also associated with a lack of sex steroid deficiency and chronic inflammation of aging 66. There appears to be an important role for Mφ in the etiology of the bone disorder. These are accompanied by increased secretion of inflammatory cytokines, including TNFα, IL-1β, and IL-6, which increase the expression of RANKL, as a TNFα superfamily member in the bone marrow micro-environment, and is necessary for osteoclastogenesis 67. Functionally, the osteogenic differentiation potential of BMSCs and the capacity for anti-inflammatory M2 polarization were notably decreases with increasing donor age 68, 69. Mφ-derived EVs, as intercellular messengers, contain several hundred proteins such as annexins, heat shock protein (HSP)-60, HSP-70, and fibronectin, and mainly affect the differentiation of BMSC, OB, and OC. The accumulation of age-related molecules packaged by EVs in the bone microenvironment could cause OP 68. Moreover, enhanced differentiation of BMSCs to adipocytes in the development of obesity, could also induces OP 70. In the obese state, the balance is clearly tilted toward the proinflammatory macrophage phenotype, which induces BMSCs differentiate toward the adipogenic lineage.

However, many of the important questions about the extent to which MSCs are influenced by Mφ-derived EVs in pathological conditions of osteoporosis in obese people will require further research to answer. Comprehensive studies of EVs derived from Mφ in OP are far worse than those in cytokines, and more research on the cargo they carry is needed, which would provide a strategy to upregulate or downregulate intercellular messengers to interfere with the function of bone-resorbing OC **(Fig. 3a)**. EVs are novel mediators in intercellular communication, and it is plausible that the modulation of EVs derived from Mφ rather than those from M2 could be a promising treatment strategy for individuals with OP.

### Bone fracture

In bone fracture therapy, good blood transport and stable fixation are the basic necessary conditions 71. Mφ and MSC-OB are the two most important cell lineages involved in fracture healing and bone remodeling 72. Bone healing is characterized by a cascade of well-regulated complex biological processes involving different cell types 73. During fracture healing, Mφ is found in the fracture site, and their depletion impairs effective healing 74. Mφ has been reported to regulate inflammation through cytokine signaling and, more importantly, endocytosis and exocytosis in acute and chronic inflammation.

MSC-OB and MC-Mφ participate in the inflammatory regulation of the fracture site **(Table 3** and** Fig. 3b)**. Bobby et al. 75 found that MC-derived EVs increased RUNX2 and BMP-2 levels, indicating the osteogenic differentiation of MSCs. BMSC-EVs regulate the expression of MC chemotactic protein-1 (MCP-1), MCP-3, and SDF-1 promote bone repair 76. Moreover, because osteogenesis and angiogenesis are closely linked, EVs promote fracture healing by regulating the entire osteogenesis-angiogenesis process or Mφ polarization-angiogenesis coupling.

The role of M2 in promoting angiogenesis and wound healing has been clarified 77. Mφ-EVs have angiogenetic potential *in vitro* and *in vivo* and could serve as a pro-angiogenic treatment for ischemic diseases 78. These studies indicate that EVs not only participate in bone matrix generation and mineralization, but also show potential as a diagnostic tool, especially in some underlying diseases such as diabetes, autoimmune disease, and tumors 79.

### Osteoarthritis (OA)

Osteoarthritis (OA) is a chronic joint disorder that results in joint pain and functional impairment 80. It is mainly characterized by cartilage degradation, synovial inflammation, subchondral bone erosion, and osteophyte formation 81. The most common risk factors for OA are age, sex, prior joint injury, obesity, genetic predisposition, and mechanical factors 82. Currently, there are no effective curative therapies for OA, and because of their extensive proliferation and differentiation capacities, BMSC-EVs represent a promising approach to OA therapy.

In addition to the cartilage protective and regenerative effects of BMSC-EVs, their role in the immunomodulatory effect of Mφ has also been demonstrated, as Mφ and other innate immune cells release inflammatory cytokines, which promote cartilage damage 71, 83. The increase in M1 is accompanied by hyperactivation of ADAMTS like 4 (ADAMTS-4) and MMP-13, and it aggravates cartilage loss, osteophyte formation, subchondral sclerosis, and periarticular weakness 84.

M2 polarization promotes articular homeostasis and regeneration in OA 9. In temporomandibular joint (TMJ) inflammation, M1-EVs transfer miR-1246 to inhibit glycogen synthase kinase β (GSK3β) and Axin2 expression and, then, upregulate IL-6, IL-8, IL-1β, and MMPs, accelerating inflammation 85. Interestingly, BMSC-EVs mediate the transformation of Mφ from the M1 to M2 phenotype to reduce joint tissue damage by inhibiting proinflammatory cytokines and releasing anti-inflammatory cytokines and transforming the percentage of CD4^+^CD8^+^ T cell subsets 86, 87. BMSC-EVs inhibit M1 production and promote M2 generation. Synovial fluid expresses lower levels of the proinflammatory cytokines, IL-1β, IL-6, and TNF-α, whereas IL-10, an anti-inflammatory cytokine, is released. TGF-β1-modified BMSC-derived EVs via miR-135b corroborate this result 88. Moreover, the 3D-printed ECM/gelatin methacrylate/exosome scaffold promoted the polarization of synovial Mφ to M2, exhibiting a potential therapy 89 **(Table 3** and** Fig. 3c)**. These studies revealed that EVs have the potential to effectively protect cartilage from degeneration and attenuate OA progression by modulating immunoreactivity, promoting the proliferation and migration of human OA chondrocytes, and regulating Mφ polarization.

### Periodontitis

Periodontal diseases are initiated by dysbiosis of the commensal oral microbiota, which leads to tooth loss and could contribute to systemic inflammation 90. Alveolar bone loss is a sign of periodontitis progression, and results from host immune and inflammatory responses to microbial challenges 91. Therefore, modulating the host inflammatory and immune cell processes is a promising strategy to rescue bone resorption in periodontitis. Mφ play an important role in the innate immune system and interact with oral pathogens to influence the balance of the oral microbial community. Furthermore, balancing the M1/M2 ratio is a novel prospect.

BMSC-EVs promote the regeneration of periodontal tissues by inhibiting the function of OCs, affecting Mφ polarization, and regulating TGF-β1 expression, thereby modulating the inflammatory immune response 45. Similarly, Zhang et al. 92 reported that BMSC-EVs decreased alveolar bone loss by regulating the expression of related cytokines and inhibiting osteoclastic bone resorption. M2-EVs could upregulate the IL-10 cytokine expression of BMSCs and BMMs via activating exosomal IL-10 mRNA to cells directly, which could promote osteogenesis while inhibiting osteoclastogenic differentiation and alveolar bone resorption 93. Therefore, the application of BMSC/Mφ-derived EVs for the regeneration of periodontal tissue is a promising treatment strategy **(Table 3** and** Fig. 3d)**.

## Controversy and future perspective

Although there are many areas of consensus regarding mechanisms of cell-cell communication via EVs in osteoimmunology, as with any rapidly growing field, there remain several challenges and areas of disagreement. It rapidly became clear that different Mφ phenotypes (M0, M1, and M2) have been proven to be involved in osteogenesis, and that M2 is the most beneficial for osteogenesis 94. However, there is still no consensus on which EVs secreted by different Mφ phenotypes promote osteogenesis the most 94. Yu et al. 84 found that the relative expression of osteogenesis genes was upregulated by M1-EVs but downregulated by M2-EVs. Conversely, Kang et al. 95 demonstrated that M0 and M2-EVs promoted repair/regeneration and M1-EVs inhibited bone repair. Mechanistically, they found similar miRNA cargo in M0 and M2 EVs and different miRNA cargo in M1 EVs. These controversial results could be partially explained by duration of action, culture conditions, and differences in techniques such as cell source, maturity of MC-Mφ, and co-culture conditions. Despite there is some debate, it is clear that EVs derived from MC and all Mφ phenotypes have the ability to regulate the osteogenesis of BMSCs, but the effectiveness of the process varies with physiological conditions and different phases through various signaling pathways that deliver different cargoes, resulting in different functions. In addition to conditioned media, intercellular communication can also be mediated through processes such as gap junctions, and autocrine and paracrine activities 32. Overall, these conflicting results reflect the complexities of the regulation of EVs on MC-Mφ and MSC-OB, especially considering that the human body and the *in vivo* environment are extremely complex.

Recently, studies have reported novel discoveries and forms of EVs in bone metabolism regulation, and EVs secreted by bacteria and the damaged brain are two examples. All bacteria produce vesicles for the selective export of toxins and other virulence factors into host cells. Vesicles provide a self-preserving membrane remodeling mechanism despite their high energy cost 96, 97. Vesicles from *Aggregatibacter actinomycetemcomitans*, the causative organism of periodontal and systemic diseases, can cross the blood-brain barrier to deliver small RNAs, which produce TNF-α 98. This observation suggests that EVs from the bone microenvironment can be delivered to the entire body and be activated. Conversely, systematic EVs influence bone homeostasis by resisting the local microenvironment. Xia et al. 99 demonstrated that injured neurons in the damaged brain, mainly in the hippocampus, release EVs to accelerate bone formation through miR-328a-3p and miR-150-5p targeting forkhead box O4 (FOXO4) or Cbl proto-oncogene (CBL), respectively.

Currently, bone metabolism is drawing considerable attention. HucMSC-EVs enhanced the shift from adipogenic to osteogenic differentiation of BMSCs and inhibited OC formation by transferring C-type lectin domain containing 11A (CLEC11A). HucMSC-EVs also improved bone formation, reduced marrow fat accumulation, and downregulated bone resorption in OVX and tail suspension-induced hindlimb disuse osteoporotic mice 100. Studies have focused on lipid metabolism and the function of glucose and amino acid metabolism in the bone microenvironment. Furthermore, the mechanism by which the metabolites are delivered is worth future investigation. More interestingly, Islam et al. found that mitochondrial DNA and mitochondrial cargo of BMSCs could be delivered to Mφ via EVs 101. These “useless” damaged mitochondria in BMSCs are “treasures” for Mφ, which can enable Mφ to express greater bioenergy 102.

The emphasis in this Review has highlight major concepts of how EVs functioning in intriguing biological links between BMSCs osteogenesis and M2 activation in middle and advanced stage of bone reconstruction/remodeling. Such interaction between Mφ and BMSCs based on EVs can summarize as a positive feedback or a vicious circle. M2 polarization promotes the osteogenic differentiation of BMSCs, increasing the number of OBs and leading to the secretion of a large number of “anti-inflammatory EVs” in benefiting to switching inflammation bone microenvironment. Conversely, BMSCs switching to adipogenesis will promote M1 differentiation, producing a large amount of “inflammatory EVs”, and that the inflammatory events may lead to fat accumulation, in turn, promoting M1 differentiation, causing inflammation. In various disease models, we must consider that BMSCs and Mφ have the potential for plasticity and multiple differentiation, in which EVs plays a significant role. Thus, as many anti-inflammatory EVs as possible are need to promote osteogenesis.

## Conclusions

Evidence of the crosstalk between MSCs and Mφ through EVs is accumulating. However, the exact role of pre-MC regulation in MSC-OB in bone regeneration and the regulation of EVs derived from OB, OC, and osteocytes on MC-Mφ warrant further investigation. Osteoimmunology plays an important role in autoimmune diseases through the crosstalk between MSC and Mφ by EVs in hematologic malignancies, osteopetrosis, inflammation, and other pathological conditions.

In this review, we focus on Mφ and BMSCs largely because of their strong differentiation ability, flexible regulatory ability, and easy accessibility. An understanding of these mechanisms would enhance the appreciation of skeletal biology and facilitate the establishment of targeted approaches to modify bone mass and develop new concepts for applying osteoimmune interaction mechanisms.

## Figures and Tables

**Table 1:** Published Studies on Biogenesis of monocyte-macrophage (MC-Mφ) lineage derived EVs in osteogenesis

Source and Kind	Methods: isolated and identified	Specific Cargo	Target cells and Genes	Function	Signaling Pathway	References
human monocytes-EVs	·The miRCURY™ Exosome Isolation Kit OR ultracentrifugation: 120,000g for 2h, filtration through a 0.1-μm filter ·TEM, Western blot (CD90, TSG101, CD63 and Hsp70)	-	·Human ATMSCs and Human BMSCs	Osteogenesis ↑	MC‐EVs→MSC various cytokines by MSCs↑ (CXC chemokines and IL-1) → expression of MMPs↑ →Osteogenesis ↑	Arjen Gebraad, 2018
human monocytes-EVs	·Centrifuged: 16,500*g* for 20 min, followed by filtration through a 0.22 µm filter ·Western blot (Hsp70, Tsg101 calnexin and Grp94), Flow cytometry (CD63, CD9, CD81) and Bioanalyzer analysis	-	·Human BMSCs	Osteogenesis ↑	Human monocytes-EVs improve the secretion of RUNX2 and BMP-2 in hBMSCs, promoting osteogenic differentiation	Karin Ekström, 2013
Mouse M0 macrophages-EVs	·Centrifuged: 2,000*g* for 30 min, and total exosome isolation reagent, then centrifuged at 10,000*g* for 60 min at 4 °C TEM, NTA, Western blot (CD81, CD63, CD9 and Alix)	-	·Mouse BMSCs	No significant influence on the proliferation of BMSCs	-	Yu Xia, 2020
Mouse M1 macrophages-EVs	·Centrifuged: 2,000*g* for 30 min, and total exosome isolation reagent, then centrifuged at 10,000*g* for 60 min at 4 °C TEM, NTA, Western blot (CD81, CD63, CD9 and Alix)	-	·Mouse BMSCs	Osteogenesis ↑	M1-EVs promoted the proliferation of BMSCs (7-day time)	Yu Xia, 2020
Mouse M2 macrophages-EVs	·Centrifuged: 2,000*g* for 30 min, and total exosome isolation reagent, then centrifuged at 10,000*g* for 60 min at 4 °C ·TEM, NTA, Western blot (CD81, CD63, CD9 and Alix)	-	·Mouse BMSCs	Osteogenesis ↓	M2-EVs impaired the proliferation of BMSCs (7-day time)	Yu Xia, 2020
M2 macrophages-EVs	·Ultracentrifugation ·TEM, DLS, flow cytometry (CD63, CD81)	miR-5106	·Mouse BMSCs ·SIK2 and SIK3	Osteogenesis ↑	M2-EVs containing miR-5106 promote osteogenic differentiation of BMSC via suppressing the expression of SIK2 and SIK3	Yuan Xiong, 2020
Mouse M2 macrophages-EVs	·MinuteTM efficient exosome precipitation reagent purchased from Inent Biotechnologies Company ·TEM, NTA, Western blot (CD81, CD63)	miR-690	·Mouse BMSCs ·IRS-1 and TAZ	Osteogenesis ↑	M2-EVs delivered miR-690 into BMSCs and increased the expression of IRS-1 and TAZ	Ziyi Li, 2021
Osteoclasts-EVs	·Ultracentrifugation: 2000g for 20 min, 20,000g for 30 min, 120,000g for 70 min at 4 °C ORTotal Exosome Isolation Kit:10,000g for 1h at 2-8 °C ·Dynamic light scattering, Western blot (HSP70, TSG101, TFIIB and LaminA/C) and Flow cytometry (CD963)	mir-214	·Osteoblast ·EphrinA2/EphA2	Osteogenesis ↓	OC-EVs containing miR-214 though ephrinA2/EphA2 ligand induced osteoblast dysfunction, and the down-regulation can be rescue by *Rab27a* small interfering RNA	Weijian Sun, 2016
Osteoclasts-EVs	·Ultracentrifugation: 300g for 10 min, 820g for 15 min, 10,000g for 5 min at 4 °C and passage through a 0.8-μm syringe filter to remove cell debris, and final centrifugation at 100,000g for 2h at 4 °C	miR-214-3p	·Osteoblast	Osteogenesis ↓	Osteoclast-derived exosomal miR-214-3p could be transferred into osteoblasts to inhibit osteoblastic bone formation	Defang Li 2016
Osteoclasts-EVs	·Ultracentrifugation: 2000g for 15 min, 12,000g for 15min at 4 °C and 100,000g for 2h at 4 °C ·TEM, NTA, Western blot (Lamin A/C, histone 3 CD81 and TSG101)	·miR-324	·Mouse BMSCs ·ARHGAP1	Osteogenesis ↑	Osteoclast-derived exosomal down-regulated ARHGAP1 in the RhoA/ROCK pathway to promote osteogenic differentiation	Mengmeng Li ang, 2021

**Table 2:** Published Studies on Biogenesis of mesenchymal stem cell-osteoblast (MSC-OB) lineage derived EVs in monocyte recruitment, polarization and function

Source	Methods: isolated and identified	Specific Cargo	Target cells and Genes	Function	Signaling Pathway	References
Rat BMSC-EVs	·Density‐gradient ultracentrifugation :2 subsequent centrifugations steps of 2500*g* for 15 minutes ·NTA, TEM and Western blot (CD63, CD81, HSP70 and Tsg101)	-	·Raw264.7 and rat peritoneal macrophages	M2 polarization ↑ M1 polarization ↓ reduced the inflammation	BMSC-EVs + LPS+ Raw264.7→NF‐κB p65 ↓ → AKT1 /AKT2 → M2 ↑, M1 ↓ →IL‐6, TNF‐α, IL‐1β ↓	Ruqin Xu, 2019
Rat BMSC-EVs	· Utral centrifuged: 300g for 10 min, 2000g for 10 min. After centrifugation, 0.22 μm Steritop™ and Amicon ultra-15 spinning Filter Unit, the liquid was centrifuged at 100,000g for 60 min ·TEM, Western blot (CD9, CD63 and CD81)	-	·HUVECs and macrophages	M1 polarization ↓ secretion of proinflammatory factors by M1 macrophages↓.	BMSC-EVs + HUVECs→ phosphorylation levels of VEGFR1 and VEGFR2 ↑, LATS1/2 and YAP1 ↓ BMSC-EVs→ M1 ↓ → TNF-α, IL-1β, IL-6, IL-8, and NOS-2 ↓	Yao Huang, 2020
Mouse BMSC-EVs	·Ultracentrifugation: 2000g for 30 min, 10,000*g* for 30 min, 100 000g for 70 min; ·Zeta View system, TEM and Western blot (CD81, TSG101, and CD9)	-	·Mouse Bone marrow-derived macrophages	M2 polarization ↑ M1 polarization ↓	BMSC-EVs→ M2 polarization ↑ →fibrocartilage and biomechanical property ↑	Youxing Shi, 2020
Mouse BMSC-EVs	·Filtration through a 0.22-μm filter and exosome isolation kit. ·TEM and flow cytometry (CD90)	miR-124-3p	·Mouse Bone marrow-derived macrophages · Ern1	M2 polarization ↑	BMSCs containing miR-124-3p + macrophage →Ern1 expression ↓ → M2 ↑	Ran Li, 2020
Rat BMSC-EVs	·Ultracentrifugation: 300g for 10 min, 2000g for 15 min, 10,000g for 30 min, and 100,000g for 70 min twice ·TEM, NTA and Western blot (CD9, CD63, TSG101, and Calnexin)	miR-31a-5p	· Osteoclasts · E2F2, SAHF	Osteoclasts numbers and function↑	miR‐31a‐5p→ E2F2 ↓ → SAHF assembly induces cellular aging→ osteoclastic numbers and function↑	Rongyao Xu, 2018
Human JMSC and BMSC-EVs	·Ultracentrifugation: 100,000g for 3 h, 3000*g* for 15 min, mixed with ExoQuick-TC, then centrifuged at 1500*g* for 30 min. Western blot (CD63 and CD81) and NTA	miR-223	·Human peripheral blood PBMC-derived macrophage pknox1	M2 polarization ↑ Enhances cutaneous wound healing	miR-223 + BMSCs→ targeting pknox1→M2 polarization ↑	Xiaoning He, 2019
Mouse Osteoblast-EVs	·Ultracentrifugation: 5000g for 10 min, 35,000g for 10 min, 100,000g for 70 min at 4 °C. ·Western blot (CD63) and TEM	-	· Osteoclasts	Osteoclasts numbers and function↑	Osteoblasts-EVs via RANKL-RANK → osteoclast formation↑	Alfredo Cappariello, 2018
Osteocyte-EVs	-	-	-	-	-	

**Table 3:** Function of bone relative or monocyte-macrophage lineage EVs in bone diseases (summarized above not be mentioned here)

Disease	Source and Kind	Specific Cargo	Function	Regulatory details	References
Osteoporosis	Mice BMSC-EVs	miR-27a	Osteogenesis↑ bone density↑ levels of bone resorption markers↓ Osteoclasts number↓	miR-27a inhibit DKK2 expression via Wnt/β-catenin pathway	Yan Wang, 2021
Osteoarthritis	Mice BMSC-EVs	-	M2 polarization ↑ M1 polarization ↓	Decrease the percentages of F4/80^+^ macrophages Down-regulate TNF-α Up-regulate IL-10	Stella Cosenza, 2017
	Rat BMSC-EVs		M2 polarization ↑ M1 polarization ↓	Decrease the expression of IL-1β, IL-6, and TNF-α, whereas IL-10 is released.	Jiyong Zhang, 2020
	Rat BMSC-EVs	miR-135b	M2 polarization ↑ M1 polarization ↓	TGF-β1 modified BMSC-EVs via delivering miR-135b up-regulate the lower levels of serum inflammatory cytokines and induce the polarization of synovial macrophages to M2 in OA rats.	Rui Wang, 2021
TMJ inflammation	Mice M1-EVs	miR-1246	Induce inflammation in condylar chondrocytes	miR-1246 inhibits GSK3β and Axin2 expression, causing activation of the Wnt/β-catenin pathway	Sisi Peng, 2021
Bone fracture	Mice BMSC-EVs	miRNAs	Promote bone repair	Up-regulate expression of monocyte chemotactic protein-1 (MCP-1), MCP-3, and stromal cell-derived factor-1, maybe via miRNA	Koichi Murata, 2016
Periodontitis	Rat BMSC-EVs	-	Inflammatory infiltration↓ Bone loss↓	Decreased TNF-α and IL-17; increase IL-10 secretion, reduce osteoclast number	Yixin Zhang, 2018
	Mice BMSC-EVs	-	Alveolar bone loss↓ Inflammatory infiltration↓ Collagen destruction↓	Regulate the function of osteoclasts and affect the macrophage polarization and TGF-β1 expression Regulate the OPG-RANKL-RANK pathway	Li Liu, 2021
	Mice M2-EVs	IL-10 mRNA	osteogenesis↑ osteoclastogenesis ↓	M2-EVs could activate the cellular IL-10/IL-10R pathway via delivering exosomal IL-10 mRNA to cells directly, regulating cell differentiation and bone metabolism.	Xutao Chen, 2022

## Abbreviations

EV: extracellular vesicle
MSC: mesenchymal stem cell
OB: osteoblast
MC: monocyte
Mφ: macrophage
ECM: extracellular matrix
BMSC: bone mesenchymal stem cell
PGE2: prostaglandin E2
TNF-α: tumor necrosis factor-α
IL: Interleukin
Arg1: arginase 1
CD206: cluster of differentiation 206
OC: osteoclast
M-CSF: macropahge colony-stimulating factor
RANKL: receptor activator of nuclear factor kappa-Β ligand
MFG-E: milk fat globule factor-E
EPHB4: ephrin type-B receptor 4
FASL: FAS-Fas ligand
NRP1: neuropilin 1
SEMA3A: semaphorin 3A
CCL2: CC motif chemokine ligand 2
CXCL8: CXC motif chemokine ligand 8
SDF-1: stromal cell-derived factor 1
VEGF: vascular endothelial growth factor
RUNX2: RUNX family transcription factor 2
BMP-2: bone morphogenic protein-2
PI3K: phosphoinositide 3-kinase
MMP: matrix metalloproteinase
BMM: bone marrow-derived macrophage
ALP: alkaline phosphatase
OCN: osteocalcin
TGF-β: transforming growth factor β
STAT3: signal transducer and activator of transcription 3
LPS: lipopolysaccharide
MAPK: mitogen-activated protein kinase
TLR4: Toll-like receptor 4
SCIRI: spinal cord ischemia/reperfusion injury
Ern1: endoplasmic reticulum to nucleus signaling 1
OSM: Oncostatin M
OVX: ovariectomized
DKK2: Dickkopf WNT signaling pathway inhibitor 2
HSP-60: heat shock protein
MCP-1: MC chemotactic protein-1
TMJ: temporomandibular joint
